# Constructing intimacy in algorithm mediated online dating: experiences of Chinese young adults

**DOI:** 10.3389/fpsyg.2026.1940482

**Published:** 2026-09-21

**Authors:** Wenqi Ji

**Affiliations:** Southeast University, Nanjing, Jiangsu, China

**Keywords:** algorithmic logic, digital society, emotional interaction, online dating, youth-young adults

## Abstract

**Introduction:**

Online matchmaking has become an increasingly common way for young people to establish intimate relationships in the digital age. Rather than serving as neutral communication tools, online matchmaking platforms actively shape mate selection, emotional interaction, and relationship development through algorithmic recommendation systems. Although research on algorithmic governance has expanded rapidly, limited attention has been paid to how algorithmic logic shapes intimate relationship construction throughout online matchmaking. This study therefore explores the mechanisms through which algorithmic logic influences young people’s online matchmaking practices and intimate relationship construction.

**Methods:**

Participants included 27 young adults with experience using online matchmaking applications (14 women and 13 men; born in the 1990s). Data were collected through the Walkthrough Method, netnography, and semi-structured interviews. Guided by the coding procedures of Grounded Theory, data were analyzed using NVivo 14 through open, axial, and selective coding.

**Results:**

Three main themes were identified, each corresponding to one research question: (a) patterns of online matchmaking practices; (b) mechanisms of algorithmically mediated intimacy construction; and (c) algorithmic perception, negotiation, and resistance. Together, these themes illustrate the dynamic interplay between algorithmic logic, platform affordances, and young people’s relational practices throughout the construction of intimate relationships.

**Discussion:**

Drawing on a sociology of technology perspective, particularly Foucault’s theory of technology and technological embodiment theory, the findings demonstrate that algorithmic logic functions as an active socio-technical force rather than merely a recommendation mechanism. This study proposes a process-oriented framework of algorithmically mediated intimacy construction, highlighting how young people continuously negotiate and reconstruct intimate relationships through interactions with platform algorithms. Future research should further examine this framework across different cultural contexts and platform environments.

## Introduction

This study brings together two growing areas of research: emotional interaction in online dating and the role of algorithmic systems in shaping intimate relationships. While both topics have received increasing scholarly attention in recent years, a significant gap remains in understanding how young adults experience emotional interaction throughout the process of algorithm-mediated online dating. Existing studies have primarily examined partner preferences, self-presentation strategies, matching outcomes, and platform governance, while relatively limited attention has been paid to the lived emotional experiences of users and the processes through which emotional connections are established, negotiated, and transformed within algorithmically mediated environments. To address this gap, the present study aims to identify the key dimensions of emotional interaction experiences among Chinese young adults and explore how algorithmic systems shape these experiences.

Online dating refers to the use of digital platforms and mobile applications to seek romantic partners, establish emotional connections, and develop intimate relationships. In recent years, online dating has become an increasingly common means of relationship formation among young adults worldwide. In China, the rapid expansion of mobile internet technologies, delayed marriage trends, and changing patterns of social interaction have contributed to the widespread adoption of online dating platforms such as Tantan, Soul, and Ergou. Through personalized recommendation systems, these platforms continuously collect behavioral data, infer user preferences, and recommend potential partners, thereby influencing opportunities for interaction and relationship development.

Previous research has suggested that online dating platforms fundamentally reshape contemporary experiences of intimacy. Studies have shown that users strategically manage self-presentation, selectively disclose personal information, and engage in impression management to enhance their attractiveness to potential partners (Bartsch and Subrahmanyam, 2015; Ellison et al., 2006; Gibbs et al., 2006; Toma and D’Angelo, 2017; Ward, 2017; Zhang et al., 2025). At the same time, online dating creates new opportunities for social connection while also generating challenges related to trust, authenticity, uncertainty, and emotional investment (Bandinelli and Gandini, 2022; Mohammed and Shaker, 2025; Wu, 2026). These findings suggest that emotional interaction in online dating is a complex social process that extends beyond simple partner matching.

Recent scholarship has increasingly recognized the importance of algorithmic systems in shaping online dating experiences. Algorithms influence which profiles become visible, how frequently users interact, and what forms of communication are encouraged. Rather than functioning as neutral technological tools, algorithms actively organize social encounters and structure the conditions under which emotional interaction occurs (Milan, 2015; Grassi, 2025). Consequently, emotional experiences in online dating emerge through continuous interactions between individual agency, platform design, and algorithmic governance (Tong et al., 2016; Elliott, 2022; Mulyanyuma et al., 2026).

Several theoretical perspectives have been developed to explain the relationship between technology and human behavior. Technological mediation theory argues that technologies actively shape human perception, experience, and action rather than simply serving as passive instruments (Verbeek, 2015). Similarly, perspectives on technological governance emphasize how digital platforms regulate users’ behavior through data collection, recommendation systems, and platform rules (Khare, 2025). These perspectives provide important insights into how emotional interaction may be shaped by algorithmic environments.

Existing studies have identified various factors associated with online dating experiences. However, several limitations remain. First, most studies focus on behavioral outcomes and matching success, while relatively little attention has been paid to the process through which emotional interaction develops under algorithmic conditions. Second, much of the existing literature relies on quantitative approaches, making it difficult to understand users’ subjective experiences and meaning-making processes. Third, despite the rapid growth of online dating platforms in China, limited research has examined the emotional experiences of Chinese young adults within algorithm-mediated dating environments. More specifically, although existing studies have examined algorithmic governance, digital intimacy, and platform-mediated relationships, less attention has been paid to how algorithmic mechanisms concretely participate in the construction, negotiation, and transformation of intimate relationships within online dating contexts.

In China, young people’s use of online dating platforms often reflects dual expectations from family and the state, with family pressure, social norms, and motivations including social recognition, romantic relationships, sexual experiences, and socializing contributing to platform use (Gao et al., 2024). State policies and media discourses further intensify anxieties surrounding marriage and relationships, making online matchmaking a process through which social norms are re-embedded through the combined influence of family, the state, and platforms (Yu, 2025; Aggleton et al., 2023). Unlike Western contexts, where online dating is often associated with individual choice and romantic freedom (Bonilla-Zorita et al., 2021), online matchmaking in China is embedded within broader social expectations concerning marriage, family responsibility, and appropriate partner selection. However, existing research on Chinese online dating has primarily emphasized cultural norms and structural influences, while the micro-level interaction between algorithmic logic and these social expectations remains insufficiently explored.

To address this gap, rather than viewing algorithms merely as technical tools that facilitate matching, this study explores how algorithmic systems participate in shaping users’ perceptions of potential partners, self-presentation strategies, emotional expressions, and relationship practices. It further examines how users recognize, negotiate, and respond to algorithmic influence within everyday online dating practices. Through this approach, the study extends technological sociology by shifting attention from traditional domains such as information, consumption, and governance toward the construction and negotiation of intimate relationships in digital society.

In the present study, participants described diverse experiences of emotional engagement, self-presentation, trust negotiation, algorithmic influence, and relationship development during online dating. These accounts highlighted the importance of examining emotional interaction as a dynamic process shaped by both individual actions and platform infrastructures. Drawing on semi-structured interviews, platform walkthroughs, and netnographic observations, this study employs grounded theory to explore emotional interaction experiences among Chinese young adults in algorithm-mediated online dating.

Grounded in a sociology of technology perspective and drawing on Foucault’s theory of technology and technological embodiment theory, the present study seeks to identify the major dimensions of emotional interaction experiences and examine how algorithmic systems influence the construction, negotiation, and transformation of intimate relationships in contemporary digital society.

## Research questions

This study seeks to explore how algorithmic logic shapes young people’s emotional interactions and intimate relationship practices on online matchmaking platforms. Specifically, it aims to understand how platform algorithms influence users’ mate selection, interactional experiences, and responses to algorithmic governance. The study addresses the following research questions:

(a) What patterns characterize young people’s online matchmaking practices under platform algorithmic logic?

(b) How does algorithmic logic shape mate selection criteria, interactional decision-making, and the development of intimate relationships during online matchmaking?

(c) How do young people perceive, negotiate, and respond to algorithmic influence throughout their online matchmaking experiences?

## Methods

### Methodological approach

In the present study, three complementary qualitative approaches were employed: the Walkthrough Method (Light et al., 2018), netnography (Kozinets, 2019), and semi-structured interviews. The Walkthrough Method enabled a systematic examination of the platform’s interface design, functional architecture, governance mechanisms, and algorithmic features. Netnography provided a contextual understanding of users’ naturally occurring interactions, self-presentation, and community practices across online matchmaking platforms and related online communities. Semi-structured interviews offered in-depth insights into young people’s lived experiences, emotional interactions, and intimate relationship practices within algorithmically mediated environments.

These three complementary approaches were particularly suitable for the aims of this study: to explore how algorithmic logic shapes young people’s intimate relationship construction on online matchmaking platforms. Informed by Foucault’s theorization of technology and technological embodiment, themes were derived inductively from the empirical data through a Grounded Theory-informed coding process and subsequently interpreted in dialogue with these theoretical perspectives. This theoretically sensitized yet inductive analytical approach ensured that the findings remained grounded in participants’ lived experiences while achieving theoretical coherence. The reporting of the interview component was guided by the Consolidated Criteria for Reporting Qualitative Research (COREQ; Tong et al., 2007).

### Participants and procedure

A total of 27 Chinese young adults born between 1990 and 1999 with experience using online matchmaking or dating applications participated in the study. Participants were recruited through purposive and snowball sampling to ensure variation in gender, age, occupation, educational background, relationship status, and platform-use experience. Recruitment was conducted through social media platforms, including Xiaohongshu, Weibo, and Douban, as well as online communities and referrals from previous participants. Eligibility was limited to individuals who had used online matchmaking applications for partner seeking, romantic exploration, or intimate relationship development. Recruitment continued until thematic saturation was achieved.

Data collection comprised three complementary components: platform walkthroughs, netnographic observation, and semi-structured in-depth interviews. During the initial stage, ErGou was selected as the primary research site. The researcher registered an account and engaged with the application as an ordinary user, systematically examining its interface design, functional architecture, recommendation mechanisms, governance practices, and user interaction pathways. The researcher also interacted with matched users to gain firsthand experience of platform-mediated interpersonal communication. Field notes, screenshots, and screen recordings were used to document the platform and user–platform interactions.

Building on the walkthrough, netnographic observation involved both participation in ErGou-related user communities and observation of discussions across other social media platforms. Within ErGou, the researcher joined user communication groups and participated in internal discussions and forums, while observing users’ self-presentation, including their attitudes, behaviors, profile settings, interests, and ways of initiating and maintaining connections with others. The researcher also followed discussions related to ErGou and online matchmaking on Xiaohongshu, Douban, and other social media platforms, and engaged in direct communication and interaction with users where appropriate. These interactions provided a more contextual understanding of users’ needs, perceptions, and experiences of ErGou and helped identify issues and topics for further exploration during the subsequent semi-structured interviews (see Table 1).

**TABLE 1:** Interview guide.

I. Algorithms and recommendation mechanisms
What are your expected goals when using this dating app?
How long have you been registered on this platform? Could you describe this platform in one sentence?
Do you understand the matching mechanism used by the dating platform you use?
Do you think the platform’s matching mechanism can accurately predict your partner preferences?
Do you think the platform’s recommendations influence your criteria for selecting a partner?
Do you adjust your partner preferences based on the platform’s recommendations?
Have you ever successfully dated someone recommended by the platform? Could you describe your experience in detail?
Do you actively adjust your profile information or preference settings on the platform to obtain different matching results?
**II. Emotional interaction mechanisms**
How do you usually meet new people on the platform?
What types of people do you tend to prioritize when choosing whom to communicate with on the platform?
Do you think interactions on online matchmaking platforms can easily generate genuine emotional connections?
Among your experiences of making connections on the platform, approximately what proportion of interactions have progressed to offline relationships?
What factors do you think play important roles in the development of relationships?
What forms of communication do you mainly use when interacting with others on the platform?
**III. Differences between online and offline emotional interaction**
Why do you choose online matchmaking rather than offline matchmaking?
Have you ever participated in offline matchmaking? What differences do you perceive between matchmaking through dating apps and offline matchmaking? Which approach do you prefer?
What differences do you think exist between online interactions on the platform and offline interactions in real-life contexts?
Have you ever encountered cases where algorithmic recommendations were inaccurate or where interactions failed?
Do you think there are differences between your online self and your offline self? If so, what are these differences?
How would you evaluate your overall experience with online matchmaking?
**IV. Basic information**
What is your age?
What is your gender?
What is your educational background?
What is your occupation?

Semi-structured interviews were then conducted with all 27 participants (see Table 2). Interviews focused on platform-use experiences, emotional interactions, partner-selection practices, perceptions of algorithmic recommendation, and intimate relationship development. Open-ended questions and follow-up prompts were used to elicit detailed accounts of participants’ experiences and interpretations. With participants’ consent, interviews were audio recorded and transcribed verbatim. All personally identifying information was removed during transcription, pseudonyms were assigned, and participants were informed of their right to withdraw from the study at any stage. All participants provided informed consent before data collection.

**TABLE 2:** General characteristics of the study participants (*N* = 27).

ID	Gender	Birth year	Education	City	Occupation
M1	Male	1996	Bachelor’s	Xi’an, Shaanxi	Freelancer
M2	Male	1999	Bachelor’s	Foshan, Guangdong	Software Engineer
F1	Female	1994	Bachelor’s	Dali, Yunnan	Software Engineer
F2	Female	1997	Bachelor’s	Qingdao, Shandong	Product/Operations
M3	Male	1999	Associate	Harbin, Heilongjiang	Student
F3	Female	1995	Bachelor’s	Shenyang, Liaoning	Administrator
M4	Male	1999	Bachelor’s	Hefei, Anhui	Government Staff
F4	Female	1999	PhD Candidate	Seoul/Zhejiang	Student
F5	Female	1993	PhD	Taizhou, Zhejiang	Teacher
F6	Female	1993	Bachelor’s	Taiyuan, Shanxi	Teacher
F7	Female	1999	Master’s	Shanghai	Accountant
M5	Male	1997	Associate	Hangzhou, Zhejiang	Chef
M6	Male	1996	Bachelor’s	Guizhou	Administrative Staff
M7	Male	1990	Master’s	Guangxi	Doctor
M8	Male	1992	Bachelor’s	Harbin, Heilongjiang	Accountant
M9	Male	1995	Bachelor’s	Hangzhou, Zhejiang	Software Engineer
M10	Male	1995	Bachelor’s	Guizhou	Police Officer
M11	Male	1995	Bachelor’s	Hubei	Logistics
M12	Male	1996	Bachelor’s	Sichuan	Software Engineer
M13	Male	1996	Bachelor’s	Zhengzhou, Henan	Software Engineer
F8	Female	1995	Bachelor’s	Guizhou	Teacher
F9	Female	1997	Bachelor’s	Guizhou	Teacher
F10	Female	1993	Bachelor’s	Quanzhou, Fujian	Homestay Owner
F11	Female	1997	Bachelor’s	Fuzhou, Jiangxi	Accountant
F12	Female	1995	Bachelor’s	Qingdao, Shandong	Sales
F13	Female	1995	Master’s	Kunming,Yunnan	Doctor
F14	Female	1993	Bachelor’s	Hangzhou, Zhejiang	Social Media Operations

### Data analysis

Data collected through the Walkthrough Method, netnographic observation, and semi-structured interviews were analyzed using Grounded Theory with the assistance of NVivo 14. Theoretical perspectives on technology and technological embodiment were used to inform the initial research framework and sensitizing concepts, rather than to impose predetermined categories on the empirical materials. Following the principles of Grounded Theory, the analysis proceeded inductively from the data to identify concepts, categories, and relationships concerning young people’s online matchmaking practices and the interaction between algorithmic logic and intimate relationship construction.

First, all empirical materials were systematically organized and repeatedly reviewed to achieve familiarity with the data. Platform walkthrough materials, including screenshots, screen recordings, and field notes documenting interactions with the application, were examined alongside netnographic materials collected from ErGou and related discussions on Xiaohongshu, Weibo, and Douban (see Table 3). The 27 semi-structured interviews were transcribed verbatim in Chinese, generating a corpus of more than 100,000 Chinese words. These materials were continuously compared throughout the analysis to identify recurring patterns and emerging concepts.

**TABLE 3:** Platform walkthrough log (selected excerpts; privacy-sensitive content omitted).

Stage	Walkthrough observations
Initial registration stage	The registration process consists of four verification steps, and the platform presents itself as a ‘serious dating platform’ from the outset.
	The platform requires real-name verification as part of the registration process.
	The platform requires users to upload a real-person profile photo for verification. During the walkthrough, I found that an image sourced from the internet could also pass the verification process, indicating a potential loophole in the verification mechanism.
	Upon completion of registration, the platform’s official assistant automatically sent reminders concerning platform rules.
Usage process	The check-in function rewards users with ‘dog food,’ the platform’s virtual currency.
	The profile setup interface prompts users to select personal tags, most of which are predefined by the platform.
	The platform prompts users to complete and supplement their profile information. Some fields are mandatory, while others are optional.
	The profile page displays an assessment of the account’s activity status.
	The platform provides multiple categories through which users can specify partner preferences and requirements.
	Within the recommendation preference settings, users can specify their preferences and requirements, while VIP membership provides access to additional filtering options.
	Values-based questions are incorporated into the platform’s matching and screening process.
Getting to know potential matches	The platform provides settings for specifying preferred partner types.
	Access to information about users who have expressed interest requires membership or payment using the platform’s ‘dog food’ currency.
	Match profiles contain multiple user tags and relatively detailed personal information.
Planet: topic discussions	The ‘Planet’ section provides topic-based discussions and functions as an additional channel for social interaction.
	The discussions cover a range of topics, with many centered on dating and marriage.
Offline activities	The platform provides dedicated offline dating and social activities.
	Matchmaking services are offered as an additional service.
	The platform provides relatively high-priced service options.
	The presentation of offline services emphasizes access to matchmaking-related resources.
Account deletion	The account management interface provides options to activate invisible mode or change one’s dating status without immediately deleting the account.
	The account deletion process asks users to indicate their reasons for leaving and provide suggestions for product improvement.

Then, Coding and analysis were conducted iteratively through open, axial, and selective coding. During open coding, meaningful segments of the data were identified, conceptualized, and labeled inductively, with participants’ own expressions and meanings retained where appropriate. This process generated 36 open codes capturing dimensions of online matchmaking motivations, behavioral practices, interactional experiences, emotional responses, and perceptions of algorithmic influence. Axial coding then examined relationships among these open codes by comparing, integrating, and organizing similar or closely related concepts into broader categories, resulting in 13 axial categories that captured connections among behavioral motivations, action pathways, algorithmic intervention, and emotional interaction. Finally, selective coding integrated the axial categories into a core analytical structure. The 13 axial categories were synthesized into four selective categories: behavioral patterns, behavioral pathways, algorithmic influence, and algorithmic resistance. These categories were integrated to construct a theoretical account of how algorithmic logic interacts with young people’s practices and emotional experiences in the construction of intimate relationships on online matchmaking platforms. Overall, the analysis generated 36 open codes, 13 axial categories, four selective categories, and 798 coded references (see Table 4).

**TABLE 4:** Coding framework illustrating themes, categories, and codes.

Main category (theme)	General category	Subcategory (representative codes)
Behavioral patterns	Romantic motivation	Family pressure
		Age anxiety
	Social motivation	Expanding social networks
		Sharing daily life
	Instrumental rationality	Pursuing efficiency
		Criteria-based screening
	Emotional experience	Emotional resonance
		Shared interests
		Companionship and communication
Action pathways	Online screening	First impressions
		Information evaluation
		Match screening
	Online interaction	Perceived authenticity
		Forms of communication
		Sense of discrepancy
	Offline transition	Authentic dating
		Conflict and tension
		Indecision
Algorithmic influence	Algorithmic front end	Visibility
		Reminders
		Pacing of communication
	Algorithmic mediation	User profiling
		Ranking
		Interaction scripts
	Algorithmic back end	Commercial capital logic
		Social structures and norms
		Cultural structures
Algorithmic resistance	Individual perception	Algorithm awareness
		Algorithmic experience
		Algorithmic reflection
	Subjective resistance	Platform configuration and manipulation
		public expression on platforms
		Switching to matchmaking platforms
	Negotiation and contestation	Algorithmic adjustment
		Self-management
		Shifting emotional needs

**TABLE 5:** Four patterns of online matchmaking practice.

Interaction orientation	Marriage-oriented motivation	Social-oriented motivation
Rational interaction	Utilitarian matchmaking	Exploratory hybrid
	Efficiency-oriented screening	Exploration with rational evaluation
	Goal: marriage	Goal: expanding opportunities
Emotional interaction	Emotional experimentation	Entertainment socializing
	Relationship testing before commitment	Emotional companionship and social interaction
	Goal: long-term relationship	Goal: companionship

Throughout the analytical process, theoretical perspectives and relevant literature were consulted as sensitizing resources to refine emerging concepts and enhance theoretical coherence, while the categories and relationships were derived primarily from the empirical data.

### Trustworthiness of the analysis

Several strategies were employed to ensure the trustworthiness of the study (Guba, 1981; Lincoln and Guba, 1986; Hsieh and Shannon, 2005). First, credibility was enhanced through semi-structured interviews with participants experienced in online matchmaking. Platform walkthrough and netnographic observations were conducted before interviews to understand the platform context and user practices, which informed the interview framework. Interview transcripts and observational materials were repeatedly reviewed to ensure accurate interpretation of participants’ experiences. Second, transferability was supported by confirming data saturation during data collection and analysis. The findings were reviewed by two sociology professors not involved in the study and one professor with expertise in ethics and sociology to evaluate their contextual applicability. Third, dependability was ensured through Grounded Theory procedures, including open, axial, and selective coding. NVivo 14 was used to manage transcripts, codes, and analytical materials, and an audit trail was maintained throughout the research process. Fourth, confirmability was strengthened through researcher reflexivity and careful verification of translated quotations against the original Chinese transcripts. Extensive verbatim quotations were used to ensure that findings remained grounded in participants’ experiences.

### Findings

The analysis generated three overarching themes that capture how algorithmic logic shapes emotional interaction throughout young people’s online matchmaking experiences. Rather than functioning solely as a technical infrastructure for partner recommendation, algorithms emerged as active socio-technical forces participating in the construction, regulation, and negotiation of intimate relationships. Across all themes, participants described emotional interaction as a dynamic process situated at the intersection of individual agency, platform affordances, and algorithmic governance. These findings are interpreted through the theoretical lenses of algorithmic governance, Foucault’s governmentality theory, emotional capitalism, and postphenomenology to illuminate the reciprocal relationship between technological mediation and intimate practices. The first theme, Patterns of online matchmaking practices and emotional interaction trajectories, examines how young people engage with online matchmaking platforms through diverse motivations, interactional orientations, and relationship expectations. It identifies different patterns of matchmaking practices and further explores how emotional interaction develops from initial online encounters to offline relationship transitions, highlighting the roles of anticipation, uncertainty, embodied communication, and relationship verification in intimacy construction. The second theme, Algorithmic shaping of emotional interaction and intimacy construction, explores how platform algorithms intervene in emotional experiences through multiple layers of technological mediation. Participants described how mechanisms of visibility allocation, recommendation, ranking, scripted interaction, and platform governance influenced mate selection, interactional rhythms, perceptions of compatibility, and the ways intimate relationships were constructed and evaluated. The third theme, Negotiating and resisting algorithmic governance, examines how young people actively interpret, negotiate, and respond to algorithmic influence. Rather than remaining passive recipients of algorithmic recommendations, participants developed various strategies of adaptation, selective engagement, and everyday resistance, demonstrating the ongoing co-construction between technological systems and user agency. Taken together, these themes reveal that emotional interaction in online matchmaking is not merely an interpersonal process but a socio-technical practice continuously shaped through the interaction between platform algorithms and individual agency. Algorithmic logic does not directly determine relationship outcomes; instead, it restructures the conditions under which intimacy is initiated, experienced, negotiated, and transformed in contemporary digital society.

### Diverse patterns of online matchmaking practices

From traditional offline forms of mediation to technologically mediated online platforms, matchmaking among young people has undergone a transition from being mediated by human networks to being mediated by technological infrastructures. Online dating platforms represented by Ergou and Qingteng Love have gradually become mainstream channels for contemporary young adults seeking romantic relationships, substantially expanding individuals’ partner-selection opportunities and possibilities for social interaction. Through embodied communicative practices, intelligent technologies construct contextualized spaces of meaning (Pustejovsky and Krishnaswamy, 2021). Accordingly, intimate relationship practices among young people on these platforms have exhibited new characteristics, including increasing dependence on media technologies and the diversification of interactional forms.

Participants described online matchmaking as a heterogeneous practice shaped by different relational goals and interactional orientations. Rather than representing a single pathway toward marriage or romantic relationships, online matchmaking functioned as a flexible socio-technical space where young people negotiated marriage expectations, social exploration, emotional experiences, and practical considerations. The analysis identified two central dimensions underlying these practices: participants’ motivations for using matchmaking platforms and their preferred modes of emotional interaction. The intersection of these two dimensions generated four patterns of online matchmaking practice: utilitarian matchmaking, entertainment socializing, exploratory hybrid, and emotional experimentation.

For some participants, online matchmaking was primarily perceived as an efficient mechanism for identifying potential marriage partners. These participants approached platforms with relatively clear relationship expectations and frequently relied on observable attributes, including educational background, occupation, income, family background, and future plans, as preliminary criteria for evaluation (Hitsch et al., 2010; Skopek et al., 2011; Tang et al., 2024).

*“I first look at education, job, and income. If these basic conditions do not match, I usually do not continue the conversation, because everyone’s time is limited…At the beginning of the conversation, I usually ask about the other person’s type of work and income level. If the gap is too large, I do not want to waste each other’s time” (M2).*

These narratives demonstrate that emotional engagement was often preceded by rational assessment. Participants did not necessarily reject emotional connection, but they tended to delay emotional investment until a certain level of practical compatibility had been established. In this process, matchmaking platforms became not only spaces for encountering potential partners but also technological environments that facilitated comparison, filtering, and efficiency-oriented decision-making. Algorithmic recommendation and profile-based matching further reinforced this logic by transforming personal characteristics into searchable and comparable information.

*“I’ll be honest with you. My family has been pressuring me quite a bit, and I also hope to find a suitable partner as soon as possible. So, I actually think it’s quite good that the platform helps me select potential partners based on certain criteria. I don’t really resist that. I mean, I can’t seriously expect to find love through some kind of home-invasion-style romance, can I? If you go and look at those offline matchmaking corners nowadays, that’s where you’ll find the real robbery.” (F1)*

This instrumental orientation was closely intertwined with family expectations and broader marriage norms (Carter, 2017). For some participants, partner selection on digital platforms remained influenced by traditional criteria associated with social stability and family approval.

*“My family hopes that I can find someone within the public sector, so I first check whether the person is a civil servant or works in a public institution. Although people say it is free love, families still care about whether the person has a stable job and what their family background is like. The screening conditions on the platform are actually similar to how parents used to ask around through acquaintances” (F12).*

These accounts suggest that digital matchmaking does not simply replace traditional relationship formation mechanisms. Instead, existing marriage expectations are translated into digital categories that can be searched, evaluated, and ranked. Through this process, online matchmaking platforms facilitate a form of platform-mediated assortative matching, in which individual choices remain embedded within broader social structures and cultural expectations (Slater, 2013; Lin and Lundquist, 2013).

*“I feel like the system assumes that I should be looking for women younger than me, as if that were simply a matter of course. Sometimes I feel like I’m not actually looking for the right person, but rather being reminded of when I should get married and what kind of person I should be looking for.” (M8)*

However, not all participants approached matchmaking platforms with immediate marriage-oriented goals. For others, these platforms represented opportunities for social exploration, emotional companionship, and expanding interpersonal networks beyond existing social circles. Rather than using platforms primarily for partner selection, these participants emphasized curiosity, communication, and the possibility of developing relationships naturally (Liu and Ma, 2024).

*“I do not use social apps because I want to get married immediately. I just want to meet people from different circles and understand their lives. I think relationships should develop naturally. I would not ask about income or property at the beginning. I care more about whether we have common topics, such as liking the same film or music. If the conversation feels good, then I may consider further contact” (M6).*

In this pattern, online matchmaking functioned less as a marriage market and more as a low-pressure social environment where intimacy could gradually emerge through everyday communication (Butler, 2004; Ahmed, 2004).

Nevertheless, social-oriented matchmaking also produced uncertainty regarding relationship boundaries and future development. Because interactions often developed through continuous online communication rather than clearly defined relationship stages, some participants experienced difficulty determining the meaning and direction of these connections (Duguay, 2017).

*“Sometimes we have really enjoyable conversations, but I don’t necessarily think about whether it could turn into a romantic relationship. It’s more about having someone there for companionship.” (F2)*

These experiences indicate that digital platforms expand opportunities for emotional connection while simultaneously creating ambiguous relational spaces. Although online interaction enables intimacy to develop through frequent communication and emotional exchange, it does not necessarily lead to stable commitment or clearly defined relationships (Gibbs et al., 2011).

*“I usually talk to several people at the same time, compare them, and then see who seems more reliable. It is impossible to talk about conditions right at the beginning, but if the conversation goes well, later I still need to see whether the practical aspects are suitable” (F3).*

The final pattern, emotional experimentation, referred to participants who maintained marriage intentions while prioritizing emotional comfort and interactional experience during relationship development. For these participants, emotional compatibility became a prerequisite for further commitment.

*“I initially just talked with him about everyday things, such as what we had eaten that day or anything interesting we had seen. Gradually, our conversations became more personal, and sometimes I would share things with him that I found difficult to talk about with people I knew in real life. But we had been talking for almost half a year and had become close enough to exchange good-morning and good-night messages every day. Neither of us dared to suggest meeting offline or defining the relationship, because we were afraid that doing so might make us lose the intimacy we already had.” (F13)*

Overall, these patterns reveal that online matchmaking is neither purely a rational selection process nor simply an emotional encounter. Instead, young people continuously negotiate between efficiency and intimacy, social expectations and personal preferences, and algorithmic structures and individual agency. Through these negotiations, matchmaking platforms become spaces where contemporary youth reconstruct meanings of romance, marriage, and emotional relationships in the digital age (Stoicescu, 2019).

Taken together, these findings suggest that online matchmaking is not a single behavioral pattern. Instead, it is a dynamic socio-technical practice in which young people move between efficiency and emotion, marriage expectations and social exploration, rational screening and affective experience. Platform algorithms do not directly determine users’ relationship choices, but they provide the conditions through which these different practices become visible, comparable, and actionable (Cheng, 2024).

### Algorithmic shaping of emotional interaction

While the previous theme illustrates how intimacy gradually develops through interpersonal interaction, this process is never independent of the platform itself. Rather than acting as a neutral intermediary that simply introduced potential partners, matchmaking platforms continuously organized whom participants encountered, how they interacted, and how relationships progressed. Participants described algorithms not as invisible technical infrastructures but as active forces that subtly shaped emotional experiences throughout online matchmaking (Bauman, 2005). Emotional interaction therefore emerged not only through communication between two individuals but also through continuous mediation by algorithmic systems (Zhang, 2025).

*“I actually feel that these apps have broadened people’s social circles. But they have also turned some people into the bear in the story of picking corn, constantly making choices and moving on to the next option. As a result, it can become difficult to fully invest in a relationship.” (M7)*

The body serves as a medium of cognition and experience. Even within virtual environments, individuals can develop a sense of presence through their embodied operations and interactions on digital platforms (Merleau-Ponty et al., 2013). Interaction in online matchmaking is not merely a form of emotional exchange at the psychological level but also an embodied practice. Ihde’s theory of embodied technology suggests that once individuals become proficient in using technologies through practical engagement, technological artifacts tend to “withdraw” and become maximally “transparent,” thereby extending and integrating into human perception while shaping how individuals experience the world. Platform algorithms in online matchmaking function as such a technological presence, subtly becoming embedded within users’ practices and influencing their perceptions and experiences of intimate relationships (Ihde, 2002).

*“I always worry that the other person might also be talking to someone else, so I’m hesitant to invest too much.” (F9)“After talking to more people, even when I meet someone I really like, I can’t help wondering whether there might be someone more suitable out there.” (M13)*

The analysis identified three interconnected dimensions through which algorithms shaped emotional interaction: the allocation of visibility, the scripting of interaction, and the governance of relationship progression. Together, these dimensions illustrate how algorithmic logic became embedded in the construction of intimacy throughout online matchmaking. Participants frequently described recommendation algorithms as determining where emotional interaction could begin. Instead of actively searching through every profile, most participants relied on algorithmically generated recommendations to identify whom they would notice and eventually contact. Consequently, opportunities for emotional interaction were conditioned by platform visibility before users exercised their own preferences (Anderson and Wood, 2021).

*“I think the recommendations were quite accurate at first. Since I didn’t have many opportunities to meet women in everyday life, it was actually helpful that the platform recommended people who matched my preferences. Sometimes I realized that I kept seeing similar kinds of people. It felt as if the platform already knew my preferences and kept recommending the same type over and over again.”(M11)*

Rather than merely recommending potential partners, algorithms structured participants’ emotional attention by deciding who became visible and who remained invisible. Participants were therefore not choosing from all possible partners but from a pool that had already been filtered by algorithmic systems. Visibility itself consequently became a form of algorithmic power, shaping both opportunities for interaction and subsequent relationship development (Bucher, 2018).

*“When I saw that someone had read my message but didn’t reply, I couldn’t help wondering whether they weren’t interested or whether the algorithm had simply stopped pushing my messages.” (F10)*

Algorithmic influence also extended beyond recommendation into the interaction process itself. Participants described platform features such as read receipts, online status indicators, matching notifications, and message reminders as continuously influencing how they interpreted one another’s behavior (Atalatti and Pawar, 2024). These seemingly ordinary interface designs gradually established implicit expectations regarding responsiveness, communication frequency, and emotional commitment (Boyd and Markowitz, 2026).

*“Sometimes the app reminds you that someone has viewed your profile or liked you. Even if you weren’t planning to message them, you become curious and end up clicking on their profile.” (F3)*

These accounts suggest that platform interfaces actively scripted emotional interaction by subtly directing users’ attention and behavioral expectations. Rather than communicating entirely on their own terms, participants increasingly adjusted their interaction according to platform-generated cues. Emotional responses therefore became intertwined with algorithmic feedback mechanisms (Church and de Oliveira, 2013; David and Cambre, 2016).

*“I actually don’t mind the platform recommending people based on my preferences because I’m hoping to find someone as soon as possible. But sometimes it also feels as though the platform intentionally keeps good matches just out of reach.”(F7)*

Finally, participants emphasized that commercial logics and platform governance continued to shape relationship development throughout platform use. Visibility was not distributed equally, and several participants believed that premium membership, profile activity, and platform engagement influenced recommendation opportunities (Gillespie et al., 2014).

*“At first I would stop scrolling once I found someone interesting. Later I kept thinking that maybe the next person would be even better. After a while, it no longer felt like meeting people but like comparing endless options.” (M11)*

Rather than simply increasing users’ choices, platform algorithms encouraged continuous comparison and repeated evaluation. Emotional interaction consequently became embedded within an environment characterized by abundance, uncertainty, and the persistent possibility of better alternatives.

*“At first, I looked forward to it every day, but later I realized that I was just constantly swiping, and gradually, I stopped feeling anything.” (F11)*

Participants simultaneously benefited from algorithmic efficiency while experiencing emotional fatigue, decision overload, and uncertainty regarding commitment (Illouz, 2012).

*“The most agonizing part is seeing that the other person has read my message but hasn’t replied. That kind of waiting makes me really anxious.” (M1)*

Taken together, these findings suggest that algorithms influence emotional interaction not by determining relationship outcomes directly but by continuously reorganizing the conditions under which intimacy is initiated, experienced, and evaluated. Recommendation systems shape visibility, interface design structures interaction, and platform governance regulates relationship opportunities (Foucault, 2023). Emotional interaction in online matchmaking should therefore be understood as a process jointly constructed through human agency and algorithmic mediation rather than interpersonal communication alone.

### Negotiating algorithmic influence in online matchmaking

Algorithms subtly shape the pathways and rhythms of emotional interaction in online matchmaking. However, individuals are not entirely passive recipients of algorithmic influence. Instead, they gradually develop an understanding of platform mechanisms and continuously respond to and adjust their behaviors throughout interactions. As Foucault argued, even when individuals exist within complex networks of power, they still retain the capacity for creativity and resistance (Foucault, 2023).

*“The longer I use the app, the more I get a sense of how it works. The system keeps recommending the same type of people, as if it has learned my habits in the background. If you ask me to explain how it does that, I honestly couldn’t say. But after I swipe right on someone I like, most of the recommendations that follow are basically the same type. Later, I deliberately started liking people from different types, just to see whether the recommendations would change. I haven’t really thought about it that much—I just chat with whoever the system recommends. But it feels like the system has already filtered people for me.” (M12)*

Users’ responses to algorithmic influence involve three interconnected dimensions: algorithmic awareness, algorithmic negotiation, and algorithmic resistance (Liu et al., 2024). Algorithmic awareness refers to users’ recognition of how algorithms shape visibility, matching, recommendation, and interaction. Algorithmic negotiation describes how users adapt their self-presentation, interaction patterns, and platform use in response to perceived algorithmic influence, including attempts to work with or around platform mechanisms. Algorithmic resistance involves more active efforts to circumvent, reject, or challenge algorithmic arrangements when they are perceived as constraining users’ autonomy or emotional choices. These dimensions are analytically distinct but interconnected, as users may move between awareness, negotiation, and resistance as their platform experiences develop (Viduchinsky, 2026). Together, these practices illustrate that users were not merely governed by algorithms but actively participated in reshaping the meaning and influence of algorithmic systems throughout online matchmaking.

A recurring finding across participants’ accounts was that algorithmic influence was neither invisible nor unquestioningly accepted. As participants accumulated experience with online matchmaking platforms, many gradually developed an awareness of how recommendation systems, platform rules, and commercial mechanisms shaped their interactions.

*“The platform is always prompting me to upgrade my membership. To be honest, the experience is pretty mediocre if you don’t pay. Without paying, it feels like you’re constantly being pushed into a corner. What makes me want to pay is the option to change my recommendation preferences. I don’t like most of the people I’m being recommended right now. But I’ve managed to resist the temptation because it’s just too expensive.”(M5)*

Rather than remaining passive recipients of algorithmic recommendations, participants continuously interpreted, negotiated, and occasionally resisted algorithmic influence in their everyday platform practices (Bonini and Treré, 2024). Their responses reflected an ongoing process of balancing technological convenience with the desire to preserve personal autonomy in intimate relationship formation (Meng, 2021).

*“Sometimes I feel like I’m not the one making the choices, but the system is deciding for me, and that makes me feel a little uneasy. But if I stop using it, I feel like I might miss out on opportunities. If I keep using it, though, I feel like I’m being led around by the system. It’s a pretty conflicted feeling.” (F6)*

Many participants explained that they gradually became aware that recommendation results were not random but reflected algorithmic learning based on their previous behaviors. Continued platform use enabled them to recognize recurring recommendation patterns and infer how the system categorized their preferences.

*“At first I thought everyone saw the same people. Later I realised that after using the app for a while, the recommendations became increasingly similar, almost as if it had already figured out what kind of person I liked.” (F8)*

Rather than viewing personalization as entirely positive or negative, participants regarded algorithmic recommendation as both convenient and limiting. While personalized recommendations reduced the effort required to search for potential partners, they also narrowed opportunities to encounter people outside participants’ established preferences.

*“I see the platform as just a tool. Whether it’s reliable or not ultimately depends on the people you meet.” (M3)*

Algorithmic awareness subsequently influenced how participants interacted with the platform. Instead of following algorithmic recommendations uncritically, many deliberately adjusted their behavior in an attempt to improve future recommendations. Some modified profile information, changed interaction frequency, or intentionally browsed different types of profiles because they believed such actions would reshape the recommendation system.

*“Sometimes I deliberately click on people who are different from my usual type because I feel the platform is becoming too predictable. If I keep liking the same kind of person, the recommendations become increasingly similar. Sometimes I intentionally change my browsing behaviour to see different people.” (M9)*

These accounts demonstrate that participants increasingly understood themselves not only as users of the platform but also as active participants in an ongoing interaction with algorithmic systems. Platform use therefore became a process of mutual adaptation in which both users and algorithms continuously adjusted to one another.

*“I prefer using incognito mode. I can look at other people’s profiles when I want to, and if I don’t want to chat, I don’t have to. At least I don’t have to constantly be noticed by others. Later, I started repeatedly changing my photos and tags, just to see whether I could get a little more exposure.” (F14)*

Participants also described various forms of everyday resistance that sought to reduce algorithmic influence over their emotional experiences. Rather than rejecting matchmaking platforms altogether, most resistance consisted of small, routine practices aimed at preserving personal autonomy. Common strategies included disabling notifications, limiting usage time, avoiding excessive profile comparison, or temporarily leaving the platform when experiencing emotional fatigue.

*“After swiping for too long, I felt I was no longer looking at people but at options. So I deleted the app for a while because I didn’t want it to influence how I judged relationships anymore. Even if the platform says someone is a highly compatible match, I still prefer to trust my own feelings after talking with them.” (F5)*

*“I do not want the system to pressure me into responding, so I deliberately wait a few hours before replying. This makes me feel that I am the one in control of the relationship.” (M10)*

Rather than rejecting technology itself, these practices represented attempts to reclaim control over emotional decision-making. Participants selectively accepted algorithmic assistance while simultaneously maintaining their own criteria for evaluating relationships (Savolainen and Ruckenstein, 2024).

*“I set a rule for myself to browse the app for only half an hour each evening. This helps me stay focused and have more meaningful conversations. Even if the system keeps recommending people to me, I still follow my own pace because I do not want it to determine how I approach relationships.” (M4)*

Taken together, these findings suggest that young people were not passive subjects governed by algorithms. Instead, they developed practical forms of algorithmic literacy through everyday platform use and continuously negotiated the boundaries between technological assistance and personal autonomy. Algorithmic influence therefore emerged as a relational process in which platform systems and human agency mutually shaped one another throughout online matchmaking.

## Discussion

This qualitative study explored how algorithmic logic shapes young people’s online matchmaking practices and intimate relationship construction in contemporary China. The findings broadly correspond with previous studies on algorithmic governance, technological mediation, and platform capitalism, which argue that algorithms are not merely technical tools for information recommendation but active socio-technical infrastructures that organize social interactions, shape users’ choices, and regulate everyday practices (Gillespie et al., 2014; Srnicek, 2017; Bucher, 2018; van Dijck and Poell, 2018). Rather than influencing only partner recommendation, the findings demonstrate that algorithmic logic permeates multiple stages of online matchmaking, including users’ motivations, interactional decision-making, relationship construction, and subsequent responses to algorithmic governance.

Beyond these perspectives, the three themes identified in this study illustrate how algorithmic influence unfolds as a continuous relational process rather than a series of isolated technological interventions. Specifically, users entered online matchmaking platforms with different relational motivations, interacted with algorithmic recommendation systems throughout relationship development, and continuously perceived, negotiated, and responded to algorithmic governance. These findings suggest that intimate relationships on digital platforms are co-produced through the dynamic interaction between algorithmic infrastructures and users’ relational practices rather than determined solely by either technological systems or individual preferences.

Building upon previous studies of online dating and algorithmic governance (David and Cambre, 2016; Duguay, 2017; Beer, 2018), this study proposes a process-oriented perspective of algorithmically mediated intimacy. Rather than conceptualizing algorithms simply as recommendation mechanisms designed to improve matching efficiency, this perspective emphasizes that algorithmic logic continuously mediates how individuals encounter potential partners, evaluate relationship possibilities, construct emotional interactions, and negotiate intimate relationships. Consequently, intimate relationship construction should be understood as an evolving socio-technical process in which platform algorithms and users’ agency mutually shape one another over time.

The first theme highlights the diversity of young people’s online matchmaking practices. Rather than adopting a single approach toward online dating, participants demonstrated different motivational orientations, interactional preferences, and relationship expectations. These findings support previous studies suggesting that online dating platforms simultaneously function as spaces for marriage, social networking, emotional companionship, and identity exploration (Finkel et al., 2012; Bonilla-Zorita et al., 2021). However, the present findings further suggest that these diverse practices are closely associated with algorithmic environments. Recommendation systems, profile displays, and interactional affordances provide different opportunities for users with different relational intentions, encouraging them to adopt distinct behavioral strategies throughout the matchmaking process.

These findings also extend previous research emphasizing individual motivations in online dating. Existing studies have primarily interpreted users’ motivations as relatively stable personal preferences. In contrast, this study suggests that motivations are continuously reshaped through interactions with algorithmic systems. Users who initially joined platforms for marriage frequently shifted toward broader social exploration, while others gradually transformed casual interactions into long-term relationship expectations. Rather than remaining fixed, motivations evolved alongside users’ experiences of algorithmic recommendation, emotional interaction, and platform participation, demonstrating the reciprocal relationship between individual agency and technological mediation.

The second theme demonstrates how algorithmic logic mediates intimate relationship construction through recommendation systems, interactional scripts, and platform governance. Consistent with previous scholarship, algorithms organize visibility by determining which users become mutually accessible and which interaction opportunities remain invisible (Walther, 1996; Gibbs et al., 2006). Consequently, mate selection is increasingly structured through algorithmically generated profiles, quantified personal information, and compatibility indicators. These technological arrangements encourage users to evaluate potential partners according to standardized and measurable criteria, thereby reinforcing efficiency-oriented decision-making during the early stages of relationship formation.

However, the findings indicate that algorithmic mediation extends beyond partner recommendation. As interactions progress, platform affordances continue to shape emotional communication, relationship pacing, and expectations of intimacy (Ellison et al., 2006; Gibbs et al., 2006). Features such as recommendation updates, profile ranking, interaction prompts, and communication interfaces influence not only whom users meet but also how relationships develop after matching. In this sense, algorithms function as relational infrastructures that organize the temporal rhythm of emotional interaction rather than merely facilitating initial encounters. This observation resonates with technological mediation theory, which argues that technologies actively participate in shaping human perception, experience, and social practice instead of serving as passive instruments (Ihde, 2002; Verbeek, 2005).

The findings further demonstrate that algorithmic mediation is not a unidirectional process but one characterized by continuous interaction between platform governance and user agency. Previous research has argued that users are not passive recipients of algorithmic recommendations but actively interpret, adapt to, and occasionally manipulate algorithmic systems (Gillespie et al., 2014; Bucher, 2018). Consistent with this perspective, participants in this study gradually developed an understanding of how recommendation algorithms operated and adjusted their behaviors accordingly. Rather than relying exclusively on algorithmic matching, they selectively modified personal profiles, altered interaction strategies, expanded platform use across multiple applications, or temporarily disengaged from recommendation systems in order to improve matching outcomes or regain control over relationship development.

These findings resonate with emerging studies on algorithmic resistance, which suggest that users continuously develop practical knowledge about algorithmic systems through everyday platform use (Karizat et al., 2021; Velkova and Kaun, 2021). However, the present study extends this literature by demonstrating that algorithmic negotiation is closely intertwined with intimate relationship practices. Participants did not resist algorithms simply because they disagreed with technological systems; rather, resistance emerged when algorithmic recommendations conflicted with emotional expectations, relationship goals, or personal understandings of intimacy. Consequently, algorithmic resistance should not be interpreted solely as opposition to technology but as an ongoing negotiation over who should retain authority in the construction of intimate relationships.

Importantly, participants rarely rejected algorithms altogether. Instead, they continuously balanced algorithmic efficiency with personal judgment. Recommendation systems were widely recognized as useful for expanding social opportunities and reducing search costs, particularly during the early stages of partner selection. At the same time, participants frequently emphasized that long-term relationship development ultimately depended upon emotional compatibility, mutual understanding, and face-to-face interaction rather than algorithmic compatibility scores. This finding supports previous studies suggesting that users often combine algorithmic recommendations with their own experiential knowledge when making relationship decisions (David and Cambre, 2016; Duguay, 2017; Finkel et al., 2012). Rather than replacing human agency, algorithms become embedded within users’ decision-making processes and are continuously evaluated, accepted, or rejected according to changing relational contexts.

Taken together, the findings suggest that intimate relationship construction on online matchmaking platforms cannot be adequately understood through either technological determinism or individual agency alone. Existing studies have often emphasized either the governing capacity of algorithms (Beer, 2018; Gillespie et al., 2014; Srnicek, 2017) or users’ strategic adaptation to digital environments (Bucher, 2018; Karizat et al., 2021). The present study demonstrates that intimate relationship construction emerges through the continuous interaction between algorithmic infrastructures, platform affordances, and users’ relational practices. Throughout online matchmaking, algorithms shape visibility, interaction, and evaluation, while users simultaneously reinterpret, negotiate, and modify these technological arrangements according to their own emotional experiences and relationship expectations.

Hence, the unique contribution of this study lies in extending current understandings of algorithmic governance beyond recommendation systems and information distribution to the domain of intimate relationship construction. Rather than conceptualizing algorithms as external mechanisms influencing isolated stages of partner selection, this study illustrates how algorithmic logic accompanies the entire process of online matchmaking, from initial motivations and interactional decision-making to emotional relationship development and subsequent negotiation with platform governance. This process-oriented perspective contributes to a more comprehensive understanding of how algorithmic governance operates within everyday intimate life.

While the findings are broadly consistent with previous studies of online dating and algorithmic governance, they point toward a more dynamic understanding of digitally mediated intimacy. Specifically, the findings highlight how online matchmaking is shaped by the continuous interaction between platform algorithms and users’ agency, rather than by either technological systems or individual preferences alone. The three themes identified in this study should therefore not be interpreted as discrete stages of online matchmaking but as interconnected processes that evolve throughout users’ relationship practices.

By integrating perspectives on algorithmic governance, technological mediation, and intimacy studies, this research proposes a process-oriented framework of algorithmically mediated intimacy construction. First, it foregrounds the relational nature of algorithmic governance by demonstrating how algorithmic influence evolves throughout different stages of online matchmaking rather than remaining confined to recommendation processes. Second, it highlights the dynamic interaction between platform affordances and users’ agency, illustrating how intimate relationships are continuously negotiated through both technological infrastructures and everyday relational practices. Finally, this framework contributes to contemporary discussions of digital intimacy by explaining not only how algorithms facilitate relationship formation but also how users actively reinterpret, negotiate, and reshape algorithmic influence throughout the construction of intimate relationships.

## Limitation

This study has several limitations. (a) Although the study included 27 young adults with diverse backgrounds and online matchmaking experiences, participants were recruited through purposive and snowball sampling. Therefore, the findings should be interpreted as providing an in-depth understanding of young people’s experiences rather than being statistically representative. (b) The findings were based on participants’ retrospective accounts of their online matchmaking experiences. As such, participants’ narratives may have been influenced by memory reconstruction and their current interpretations of past experiences. (c) Participants used different online matchmaking platforms, but the study did not systematically compare how different platform architectures and recommendation systems influenced intimacy construction. Future research could further explore platform-specific differences. (d) Finally, the study focused on young adults in the Chinese socio-cultural context and examined algorithmic mediation primarily from users’ perspectives. Future research should investigate different cultural contexts and incorporate platform-side perspectives to further validate the proposed framework.

## Recommendations for practice and future studies

The findings highlight how algorithmically mediated online matchmaking reshapes young people’s experiences of intimacy and relationship formation. This has important implications for platform governance, digital literacy education, and public policy. For instance, online matchmaking platforms should improve the transparency of recommendation systems by providing users with clearer explanations of why potential partners are recommended and offering greater control over personalization settings. Platform designers should also consider developing interaction mechanisms that better accommodate diverse relationship goals rather than assuming that all users primarily seek marriage. In addition, reducing excessive comparison cues and optimizing interface designs that encourage healthy communication may help alleviate emotional fatigue and improve users’ relationship experiences.

The findings also suggest the importance of strengthening algorithmic literacy among young people. Universities, educators, and relevant public institutions could incorporate algorithmic literacy into digital literacy education, helping young people better understand how recommendation systems, platform interfaces, and commercial mechanisms influence emotional interaction and relationship decision-making. Future research should further examine how algorithmically mediated intimacy varies across different cultural contexts, age groups, and platform environments. Comparative and longitudinal studies integrating users’ experiences with platform-side perspectives would further advance understanding of the dynamic interaction between algorithmic systems and intimate relationship construction.

## Data Availability

The raw data supporting the conclusions of this article will be made available by the authors, without undue reservation.
