# Supplementary material for: Constructing intimacy in algorithm mediated online dating: experiences of Chinese young adults
Source: Front Psychol. 2026 Sep 21;17:1940482. doi: 10.3389/fpsyg.2026.1940482 (PMC13635324; doi:10.3389/fpsyg.2026.1940482)
Supplement: Supplementary file 1 [file Supplementary_file_1.docx]

**APPENDIX 1 Consolidated criteria for reporting qualitative studies (COREQ)**

| Paper Section/  Topic | Item | Description | Reported? | |
| --- | --- | --- | --- | --- |
|  |  |  |  | Pg.# |
| Domain 1: Research team and reflexivity | | | | |
| Personal Characteristics | | | | |
| Interviewer/  facilitator | 1 | Which author/s conducted the interview or focus group? | √ | P4 |
| Credentials | 2 | What were the researcher’s credentials? e.g., PhD, MD | √ | P4 |
| Occupation | 3 | What was their occupation at the time of the study? | √ | P4 |
| Gender | 4 | Was the researcher male or female? | NA |  |
| Experience and training | 5 | What experience or training did the researcher have? | √ | P3 |
| Relationship with participants | | | | |
| Relationship established | 6 | Was a relationship established prior to study commencement? | √ | P4 |
| Participant knowledge of the  Interviewer | 7 | What did the participants know about the researcher? e.g., personal goals, reasons for doing the research | √ | P4 |
| Interviewer characteristics | 8 | What characteristics were reported about the interviewer/facilitator? e.g., bias, assumptions,  reasons and interests in the research topic | √ | P4 |
| Domain 2: study design | | | | |
| Theoretical framework | | | | |
| Methodological orientation and  theory | 9 | What methodological orientation was stated to underpin the study? e.g., grounded theory,  discourse analysis, ethnography, phenomenology, content analysis | √ | P3/P4 |
| Participant selection | | | | |
| Sampling | 10 | How were participants selected? e.g., purposive, convenience, consecutive, snowball | √ | P4 |
| Method of approach | 11 | How were participants approached? e.g., face-to-face, telephone, mail, email | √ | P4 |
| Sample size | 12 | How many participants were included in the study? | √ | P4 |
| Non-participation | 13 | How many people refused to participate or dropped out? Reasons? | √ | P4 |
| Setting | | | | |
| Setting of data collection | 14 | Where was the data collected? e.g., home, clinic, workplace | √ | P5 |
| Presence of non-participants | 15 | Was anyone else present besides the participants and researchers? | √ | P5 |
| Description of sample | con | What are the important characteristics of the sample? e.g., demographic data, date | √ | P5-6 |
| Data collection | | | | |
| Interview guide | 17 | Were questions, prompts, and guides provided by the authors? Was it used as a pilot test? | √ | P4-5 |
| Repeat interviews | 18 | Were repeat interviews carried out? If yes, how many? | NA |  |
| Audio/visual recording | 19 | Did the research use audio or visual recording to collect the data? | √ | P5 |
| Field notes | 20 | Were field notes made during and/or after the interview or focus group? | √ | P5 |
| Duration | 21 | What was the duration of the interviews or focus group? | √ | P5 |
| Data saturation | 22 | Was data saturation discussed? | √ | P5 |
| Transcripts returned | 23 | Were transcripts returned to participants for comment and/or correction? | √ | P5 |
| Domain 3: analysis and findings | | | | |
| Data analysis | | | | |
| Number of data coders | 24 | How many data coders coded the data? | NA |  |
| Description of the coding tree | 25 | Did the authors provide a description of the coding tree? | √ | P10-11 |
| Derivation of themes | 26 | Were themes identified in advance or derived from the data? | √ | P4-11 |
| Software | 27 | What software, if applicable, was used to manage the data? | √ | P6 |
| Participant checking | 28 | Did participants provide feedback on the findings? | NA |  |
| Reporting | | | | |
| Quotations presented | 29 | Were participant quotations presented to illustrate the themes/findings? Was each  quotation identified? e.g., participant number | √ | P4-11 |
| Data and findings consistent | 30 | Was there consistency between the data presented and the findings? | √ | P4-11 |
| Clarity of major themes | 31 | Were major themes clearly presented in the findings? | √ | P4-11 |
| Clarity of minor themes | 32 | Is there a description of diverse cases or a discussion of minor themes? | √ | P10-11 |
